# Altered brain functional connectivity in vegetative state and minimally conscious state

**DOI:** 10.3389/fnagi.2023.1213904

**Published:** 2023-06-29

**Authors:** Yi Yang, Yangyang Dai, Qiheng He, Shan Wang, Xueling Chen, Xiaoli Geng, Jianghong He, Feng Duan

**Affiliations:** ^1^Department of Neurosurgery, Beijing Tiantan Hospital, Capital Medical University, Beijing, China; ^2^Chinese Institute for Brain Research, Beijing, China; ^3^Beijing Institute of Brain Disorders, Beijing, China; ^4^China National Clinical Research Center for Neurological Diseases, Beijing, China; ^5^Tianjin Key Laboratory of Brain Science and Intelligent Rehabilitation, College of Artificial Intelligence, Nankai University, Tianjin, China; ^6^Department of Information and Communications Engineering, School of Engineering, Tokyo Institute of Technology, Yokohama, Kanagawa, Japan

**Keywords:** DoC, MCS, VS, functional connectivity, network representation

## Abstract

**Objectives:**

The pathological mechanism for a disorder of consciousness (DoC) is still not fully understood. Based on traditional behavioral scales, there is a high rate of misdiagnosis for subtypes of DoC. We aimed to explore whether topological characterization may explain the pathological mechanisms of DoC and be effective in diagnosing the subtypes of DoC.

**Methods:**

Using resting-state functional magnetic resonance imaging data, the weighted brain functional networks for normal control subjects and patients with vegetative state (VS) and minimally conscious state (MCS) were constructed. Global and local network characteristics of each group were analyzed. A support vector machine was employed to identify MCS and VS patients.

**Results:**

The average connection strength was reduced in DoC patients and roughly equivalent in MCS and VS groups. Global efficiency, local efficiency, and clustering coefficients were reduced, and characteristic path length was increased in DoC patients (*p* < 0.05). For patients of both groups, global network measures were not significantly different (*p* > 0.05). Nodal efficiency, nodal local efficiency, and nodal clustering coefficient were reduced in frontoparietal brain areas, limbic structures, and occipital and temporal brain areas (*p* < 0.05). The comparison of nodal centrality suggested that DoC causes reorganization of the network structure on a large scale, especially the thalamus. Lobal network measures emphasized that the differences between the two groups of patients mainly involved frontoparietal brain areas. The accuracy, sensitivity, and specificity of the classifier for identifying MCS and VS patients were 89.83, 78.95, and 95%, respectively.

**Conclusion:**

There is an association between altered network structures and clinical symptoms of DoC. With the help of network metrics, it is feasible to differentiate MCS and VS patients.

## 1. Introduction

Every year, more than 50 million people in the world suffer a variety of brain injuries (Maas et al., 2017; Jiang et al., 2019). After an acute coma, some of them fall into a disorder of consciousness (DoC) (Bernat, 2006; Fernandez-Espejo and Owen, 2013). DoC is a serious brain dysfunction that results in the patients partially or totally losing the ability to perceive the surrounding environment and their state. To maintain the life of DoC patients, long-term treatment and heavy nursing burden have brought great pressure on families and society (Bernat, 2006).

Consciousness consists of two components: arousal and awareness. Numerous studies have shown that DoC is characterized by disruptions in the neural pathways that maintain arousal and awareness. However, the pathological mechanism of DoC is still not fully known. According to the level of consciousness, DoC can be mainly classified as coma, vegetative state (VS) or unresponsive wakefulness syndrome, minimally conscious state (MCS), etc. VS patients resume the sleep–wake cycle and open their eyes spontaneously or under stimulation, but they have no obvious signs of consciousness. MCS patients show a weak and unstable state of consciousness.

Consciousness cannot be measured directly. At present, traditional behavioral scales (Giacino et al., 2004; Teasdale et al., 2014) are still the gold standard for assessing the state of consciousness. However, due to subjective differences and interference of other factors (e.g., medical complications, motor deficits, and sensory deficits) (Giacino et al., 2009), 40% of MCS patients may be misdiagnosed as VS patients based on behavioral assessment (Wang et al., 2017; Galiotta et al., 2022). This may raise serious medical and social–ethical problems. Therefore, it is urgent to seek a more effective diagnostic method for the level of consciousness.

The brain is a complex system. The network connectivity pattern of multiple brain areas interacting with each other has been proven to be the physiological basis of information processing in the brain (van den Heuvel and Sporns, 2019). Based on graph theory, this pattern is abstractly represented as a model composed of numerous nodes and edges connecting these nodes, to facilitate the understanding of the relationship between functional integration, functional separation, and human cognition (Lynn and Bassett, 2019). The default mode network was involved in the regulation of internal consciousness (Scalabrini et al., 2022). Previous studies have reported that there is a decrease in functional connectivity of the default mode network and the reduction is proportional to the degree of consciousness impairment when consciousness-related neuronal activity is severely disrupted (Vanhaudenhuyse et al., 2010; Fernandez-Espejo et al., 2012; Soddu et al., 2012). A similar phenomenon is present in the executive control network, which is involved in maintaining external consciousness (Vanhaudenhuyse et al., 2011). In addition, a thalamocortical connectivity breakdown is observed in DoC patients and is thought to be an important factor in triggering DoC (Weng et al., 2017). Ample evidence supports differences in brain connectivity patterns between DoC patients and healthy populations. Recently, the changes in brain network topology induced by impaired consciousness have been investigated in some studies. Achard et al. showed that the hub nodes in comatose patients exhibit an extensive readjustment (Achard et al., 2012). Several studies indicated that compared with normal subjects, impaired consciousness leads to a decrease in the information integration ability of the brain network (Crone et al., 2014; Chennu et al., 2017; Rizkallah et al., 2019). However, these studies were almost always conducted based on binary brain networks. Compared with the binary network, the weighted network can more realistically describe the connectivity characteristics between brain areas. The global and local topological characteristics of the weighted brain network for MCS and VS patients have not been clearly investigated. Furthermore, few studies have discussed whether network representations of MCS and VS patients are expected to be valid neural markers to distinguish them.

In this study, relying on resting-state functional magnetic resonance imaging (fMRI), we constructed the weighted brain functional networks for the normal control (NC) group, VS group, and MCS group and analyzed the corresponding structural characteristics of the brain network. Then, the network characteristics of MCS and VS patients were fed into a support vector machine to verify whether the network representations could provide useful information to effectively differentiate the two types of patients.

## 2. Materials and methods

### 2.1. Subjects

One hundred and four patients with DoC were recruited from Beijing Tiantan Hospital, Capital Medical University, China. Each patient suffered severe brain injury for more than 1 month. Based on the currently most recognized Coma Recovery Scale—Revised (CRS-R) assessment, these patients were divided into a group of 80 VS patients and a group of 24 MCS. patients by performing multiple evaluations within 2 weeks. None of the patients had any contraindications to MRI scans, and no sedation or anesthesia was performed before MRI acquisition. Due to the excessive head motion (translation > 3 mm or rotation > 3°) and terrible image quality, the data of 40 VS and 5 MCS patients were discarded in the follow-up analysis. The specific demographic and clinical information for the remaining 40 VS (age = 42.95 ± 13.90, 17 men) and 19 MCS patients (age = 40.05 ± 14.80, 11 men) is listed in Supplementary Table S1. Age- and sex-matched 30 healthy volunteers (age = 41.24 ± 11.27, 15 men) were included in the NC group. All healthy subjects have no history of neurological or psychiatric disease. The overall experimental protocol complies with the Declaration of Helsinki and is approved by the local ethics committee. Before data collection, each healthy subject and the legal surrogate of each patient were given a complete description of the experiment and signed written informed consent.

### 2.2. Data acquisition and preprocessing

All images were collected using a Discovery MR750 3.0-T scanner (General Electric, Milwaukee, Wisconsin, USA) equipped with an eight-channel head coil. fMRI images were obtained axially using a T2-weighted echo-planar imaging sequence sensitive to the blood oxygenation level-dependent contrast. The detailed acquisition parameters were as follows: repetition time (TR)/echo time (TE) = 2000/30 ms, thickness/gap = 4.0/0.6 mm, matrix size = 64 × 64 × 39, voxel size = 3.75 × 3.75 × 4 mm^3^, and flip angle = 90°. The scan time lasted 7 min, generating a total of 210 volumes. T1-weighted high-resolution structural images were acquired for registration of functional images using a sagittal brain volume imaging sequence with the following parameters, TR/TE = 8.16/3.18 ms, thickness/gap = 1.0/0 mm, matrix size = 256 × 256 × 188, voxel size = 1 × 1 × 1 mm^3^, and flip angle = 7°. During data acquisition, each participant was instructed to keep their eyes closed, lie motionless, and not think about anything. Head movements were minimized using wedge-shaped foam padding.

Data preprocessing is an essential step to reduce the noises of physiological and instrument sources and increase the signal-to-noise ratio. In the present study, data were screened and preprocessed by running Data Processing and Analysis for Brain Imaging (DPABI) Version 6.0 210501 software package (Yan et al., 2016) on MATLab R2019a. In the initial stage of data acquisition, the data quality was affected by the inhomogeneity of the magnetic field and the inadaptability of subjects. Usually, the first 10 volumes of each subject were removed to improve the accuracy of the recording. Then, slice timing correction was performed on the remaining volumes to rectify temporal offsets between slices. Each volume was realigned with the reference volume such that the spatial position of the brain corresponds exactly. Subsequently, the structural image was co-registered with the mean functional image. Gray matter, white matter, and cerebrospinal fluid were segmented from the co-registered structural image. To reduce the effect of non-neuronal fluctuations, brain white matter signals, cerebrospinal fluid signals, global signals, and head motion parameters were regressed from fMRI data. Finally, the functional images were spatially normalized to the standard space of the Montreal Neurological Institute, thus eliminating individual differences in brain size and morphology among different subjects. The voxel size was resampled to 3 × 3 × 3 mm^3^. A 4-mm full-width at half maximum Gaussian kernel was applied to smooth the spatially normalized image. The time series of all voxels were bandpass filtered between 0.01 to 0.1 Hz.

### 2.3. The construction of the brain network

In essence, the brain function network can be regarded as a model with a large number of network nodes and connection edges connecting these nodes. Here, a widely used brain partition scheme named Automated Anatomical Labeling (AAL) atlas was employed, which parcellates the brain excluding the cerebellum into 90 separated brain functional areas (45 in each brain hemisphere). The 90 areas are abstracted into network nodes. Then, the average time series of all voxels in each brain area were extracted. The relationship between the network nodes for each subject was revealed by a 90 × 90 matrix, which was obtained from calculating the Pearson correlation coefficient *r* of the time series. To improve the normality of the data, the Pearson correlation coefficient *r* was subjected to Fisher's *r*-to- *z* transformation. Owing to poor knowledge of its complexity, the negative correlation coefficients are usually not considered to establish the connection edges. Consistent with previous studies, each negative element in the matrix was set to 0. Network sparsity refers to the ratio of the actual number of connection edges to the maximum possible number of connection edges in the network. A small network sparsity means that there are a lot of isolated network nodes. A big network sparsity may introduce a batch of false connections. The first leading elements in the matrix were retained to build the connection edges between the brain areas with the strongest coupling. As a result, a total of 36 weighted adjacency matrices from the network sparsity range of 5% to 40% with an interval of 1% were generated in the graph formation.

### 2.4. The measures of brain network

Ten metrics were adopted to characterize the topological organization of the brain function network for healthy subjects, VS patients, and MCS patients, including global efficiency, local efficiency, clustering coefficient, characteristic path length, nodal efficiency, nodal local efficiency, nodal clustering coefficient, degree centrality, betweenness centrality, and eigenvector centrality. The first four are called global network metrics. The last six are referred to as local network metrics. The subsequent discussion was carried out in the weighted form of all network measures. Nodal efficiency reflects the difficulty for a node to transmit information to its neighbor. Nodal local efficiency describes the ability of information exchange between the first-hop neighbors of a node. Nodal clustering coefficient of a node quantifies the clustering degree for its first-hop neighbors. Nodal centrality reveals the importance of nodes in the whole network. Degree centrality evaluates node importance based on edge weight. Betweenness centrality assesses the status of nodes according to their contribution to information flow in the network. The eigenvector centrality of a node emphasizes the quantity and quality of its neighbors. In other words, if a node has a large number of important neighbors, the node is also very important. The characteristic path length is the average shortest path length of all node pairs. Global efficiency is defined as the average nodal efficiency of all nodes. These two indicators represent the capability of information transmission at the network level, depicting the functional integration from the side. Similarly, local efficiency and clustering coefficient are the average representations for nodal local efficiency and nodal clustering coefficient of all nodes, respectively. In addition to information interaction, they measure functional separation to some extent.

### 2.5. Embedded feature selection and L_1_ regularization

Combining the selected complex network measures, the length of the final feature vector for each subject was 544. Feature selection is a very important process in the classification task, which optimizes the original feature set by eliminating redundant features. Feature selection helps to improve the classification performance and, at the same time, reduces the training time and storage requirements. Common feature selection can be roughly divided into filtered type, wrapped type, and embedded type. The filtered type relies on the importance score of the feature to filter the features that meet the conditions, independent of the training process of the classifier. The wrapped type is closely linked to the training process. This method treats classification performance as an evaluation criterion and searches for the optimal subset of features by multiple iterations. Unlike the previous two approaches, the embedded type integrates the feature selection within the training process, enabling automatic and simultaneous feature selection during the training process. Therefore, the features were selected in this study using an embedded method (Zheng et al., 2022). Based on the logistic regression, it is mathematically described as follows.

Given the data set D = {(*x*_1_, *y*_1_), (*x*_2_, *y*_2_), ⋯ , (*x*_*m*_, *y*_*m*_)}, where *x* ∈ *R*^*d*^, *y* ∈ *R*, the loss function of the logistic regression is


$$\begin{array}{l} J\left(\omega \right)=-\overset{m}{\underset{i=1}{\sum }}\left[y_{i}\ln\;h_{\omega }\left(x_{i}\right)+\left(1-y_{i}\right)\ln\;\left(1-h_{\omega }\left(x_{i}\right)\right)\right], \end{array}$$

(1) $$\begin{array}{l} h_{\omega }\left(x_{i}\right)=\frac{1}{1+e^{-\omega^{T}x_{i}}} \end{array}$$

where ω denotes the model parameter to be learned. Due to the small sample size and excessive features, L_1_ regularization is introduced to prevent the logistic regression from falling into overfitting. The new optimization objective becomes


(2) $$\begin{array}{l} \text{min}j\left(\omega \right)\;+\text{ λ }{∥\;\omega \;∥}_{1} \end{array}$$

where λ represents the regularization parameter, λ > 0 Solve for the vector ω. Many components of the vector ω are 0. The attributes corresponding to its non-zero components are the features we need.

### 2.6. Synthetic minority oversampling technique

The sample size of the VS group was approximately twice as large as that of the MCS group. Hence, the upcoming classification task suffers from a severe class imbalance. In general, machine learning algorithms show excellent performance when dealing with class-balanced data. Class imbalance leads to the classifier learning a priori information of sample proportion. Thus, the actual prediction is focused on the majority class. To solve this problem, the SMOTE algorithm (Chen et al., 2022) was introduced in this study. The basic idea of the algorithm is to give a classifier the ability to learn features of different classes equally by synthesizing samples for minority classes. The implementation process is as follows:

(a) The distance between each sample and the other samples was calculated to obtain the *k* nearest neighbors for each sample in the minority class,
(b) The sampling rate *N* is set according to the imbalance ratio of the sample,
(c) One sample is randomly selected from the *k* nearest neighbors of each sample for linear interpolation, and the new sample is synthesized as

(3) $$\begin{array}{l} x_{new}=x_{i}+\delta \left({\hat{x}}_{i}-x_{i}\right) \end{array}$$

where *x*_*new*_ is the new sample synthesized, *x*_*i*_ is a sample of the minority class, ${\hat{x}}_{i}$ is a randomly selected sample from the *k* nearest neighbors, δ is a random factor in the range (0, 1).

(d) The process is repeated *N* times.

### 2.7. Validation and evaluation

**Figure 1:**
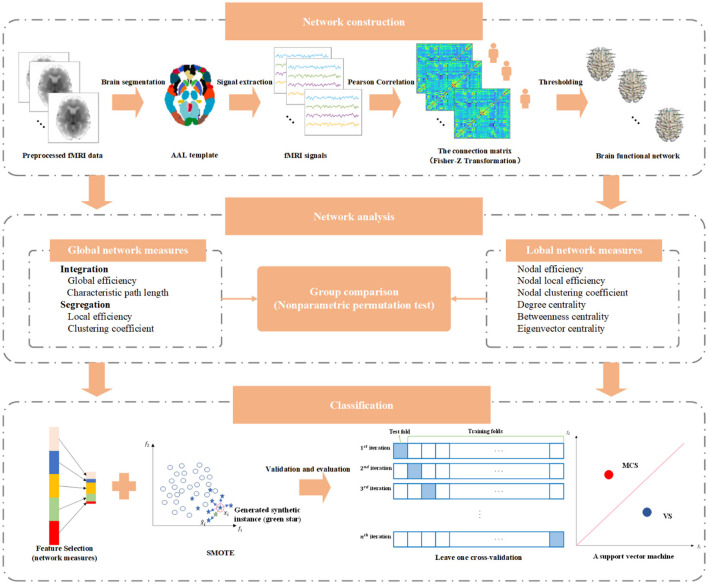
The main implementation pipeline of this study.

After feature selection and oversampling, a support vector machine was employed to identify MCS and VS patients. Usually, a small number of samples for training and testing may result in low robustness and generalization of the model. To obtain a stable and reliable performance evaluation, cross-validation is an essential step. Here, leave-one-out cross-validation was used. In each iteration, the method selects one sample as the test data, while the rest are classified as training data. Until each sample has acted as test data once, the cross-validation is over. Owing to the class imbalance, it is not reasonable to adopt accuracy alone to describe model performance. Therefore, sensitivity and specificity, two common metrics, were also complemented to evaluate the classification performance. The formula for each indicator is listed below,


(4) $$\begin{array}{l} Accuracy=\frac{TP+TN}{TP+TN+FP+FN} \end{array}$$

(5) $$\begin{array}{l} Sensitivity=\frac{TP}{TP+FN} \end{array}$$

(6) $$\begin{array}{l} Specificity=\frac{TN}{TN+FP} \end{array}$$

where *TP* denotes the number of MCS patients correctly predicted, *TN* represents the number of VS patients correctly predicted, *FP* is the number of VS patients incorrectly identified as MCS patients, and *FN* symbolizes the number of MCS patients incorrectly identified as VS patients. The main implementation pipeline of this study is illustrated in Figure 1.

## 3. Results

**Figure 2:**
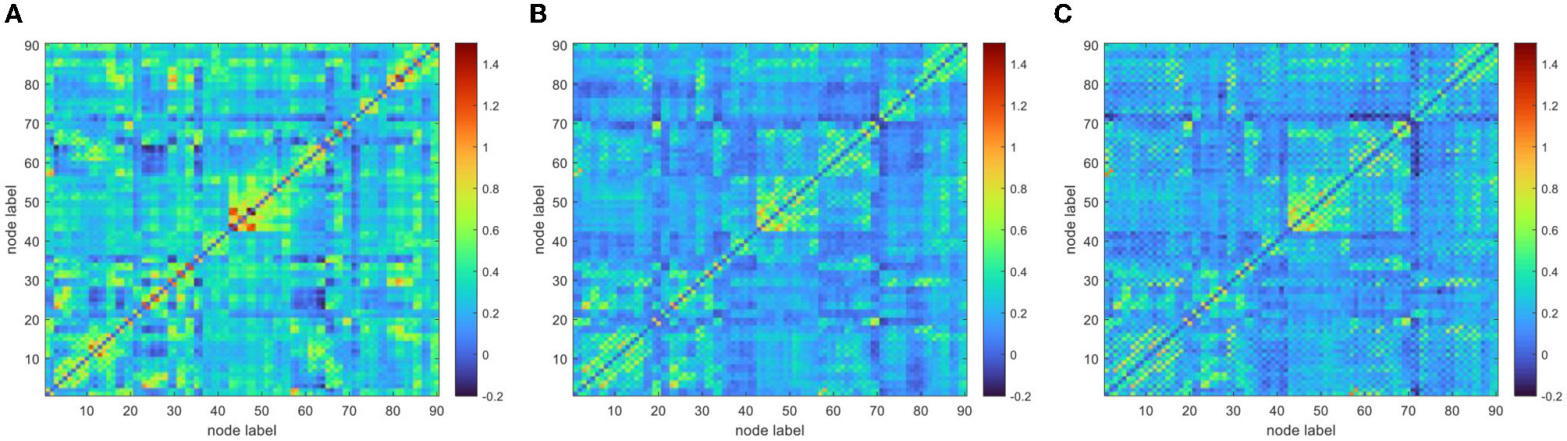
Group average connection matrix **(A)** NC group, **(B)** MCS group, **(C)** VS group.

**Figure 3:**
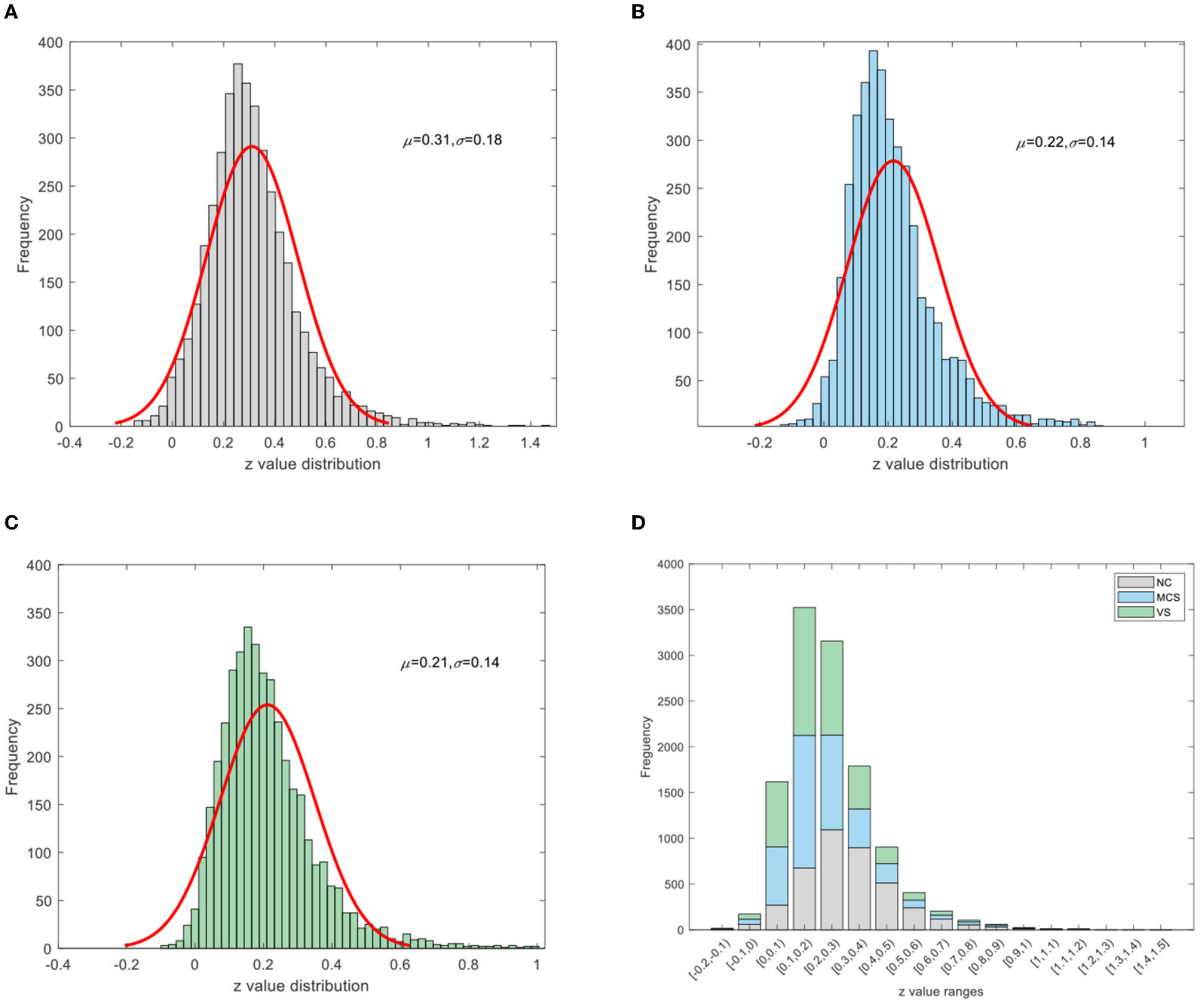
Z-value distribution **(A)** NC group, **(B)** MCS group, **(C)** VS group, **(D)** stacking diagram of the three groups.

To improve the normality of the Pearson correlation coefficient, a non-linear correction to the correlation coefficient matrix was performed using Fisher's *r*-to-*z* transformation. The group-averaged corrected correlation coefficient matrix is shown in Figure 2, where the horizontal and vertical axes are the node (brain area) labels. Each element in the matrix portrays the coupling relationship between the corresponding two brain regions. From Figure 2, it can be roughly inferred that the average connection strength of the NC group was higher than that of the patient group. The group-averaged *z*-value distribution was estimated with the help of histograms to confirm this hypothesis, as shown in Figure 3. Because of symmetrical characteristics, only the elements in the upper triangular region of the matrix were investigated. Quantitatively, the group-averaged *z*-values of the NC group, MCS group, and VS group roughly followed a normal distribution with means μ of 0.31, 0.22, and 0.21 and standard deviation σ of 0.18, 0.14, and 0.14, respectively. The group-averaged *z*-values of the MCS group and VS group were mainly concentrated in the interval (0, 0.6). The number of *z*-values >0.3 was reduced in the MCS group and VS group compared with the NC group. Therefore, when we constructed the brain functional network using the first 5% to 40% of *z*-values with an interval of 1%, the average connection strength of the NC group was necessarily higher than that of the remaining two groups. In addition, Figures 2, 3 show that the average connection strength was roughly equivalent in the MCS and VS groups.

**Figure 4:**
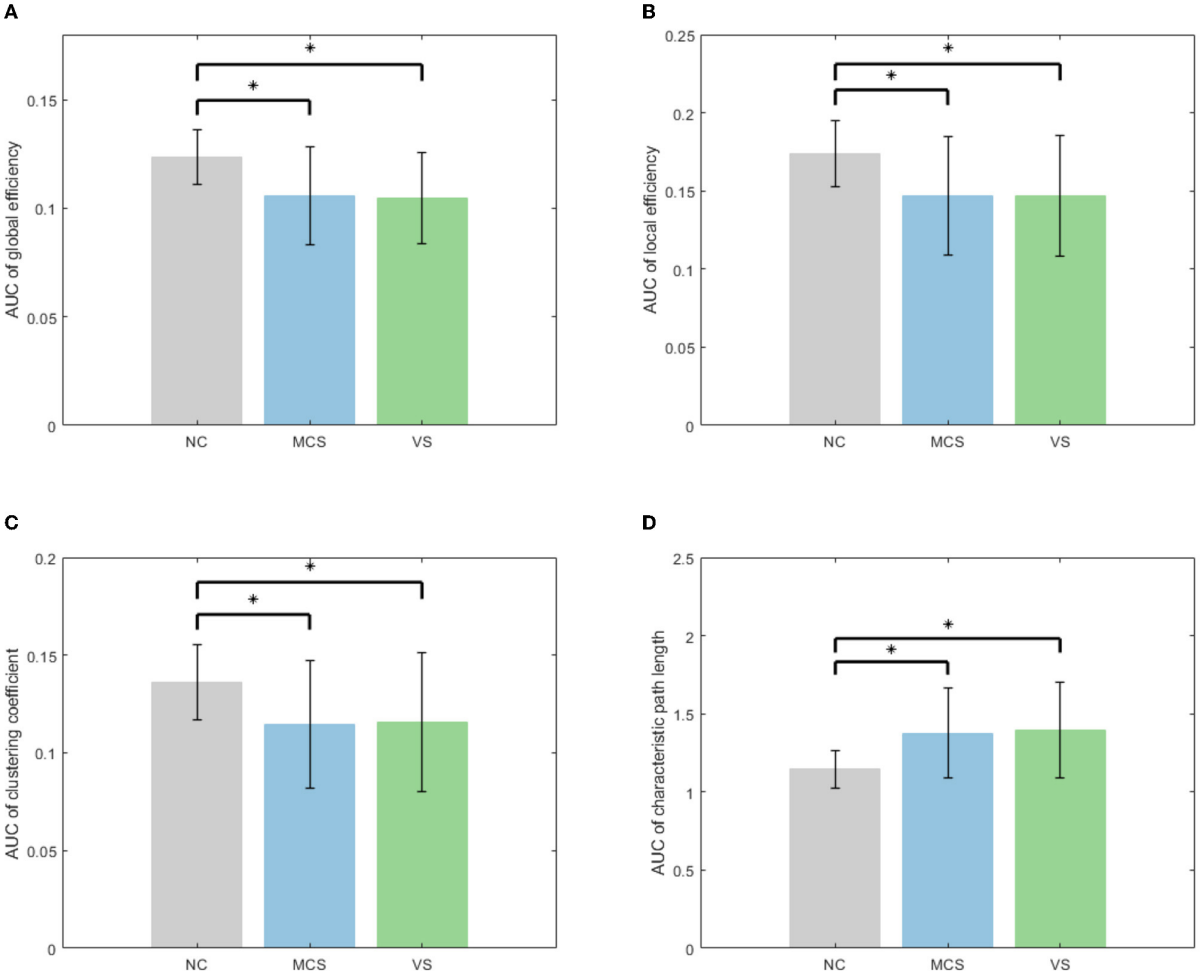
Between-group comparison of global network measures **(A)** AUC of global efficiency, **(B)** AUC of local efficiency, **(C)** AUC of clustering coefficient, and **(D)** AUC of characteristic path length.

Subsequently, the area under the curve (AUC) for each network measure was calculated. The AUC of the network measure is a summary scalar reflecting the structure of the brain network. With this approach, the network parameters of various thresholds can be integrated. A non-parametric permutation test was applied to assess the between-group difference in the AUC of each network measure. The number of permutations was 10,000. The significance level α was 0.05. The between-group comparison of global network measures for the NC group, MCS group, and VS group is given in Figure 4. The significant differences between groups are marked with an asterisk. As shown in Figure 4, the global efficiency, local efficiency, and clustering coefficient of the MCS group and VS group were significantly lower than those of the NC group (*p* < 0.05). The characteristic path length of the MCS and VS groups was significantly higher than that of the NC group (*p* < 0.05). However, there was no difference in these measures between the MCS and VS groups (*p* > 0.05).

**Figure 5:**
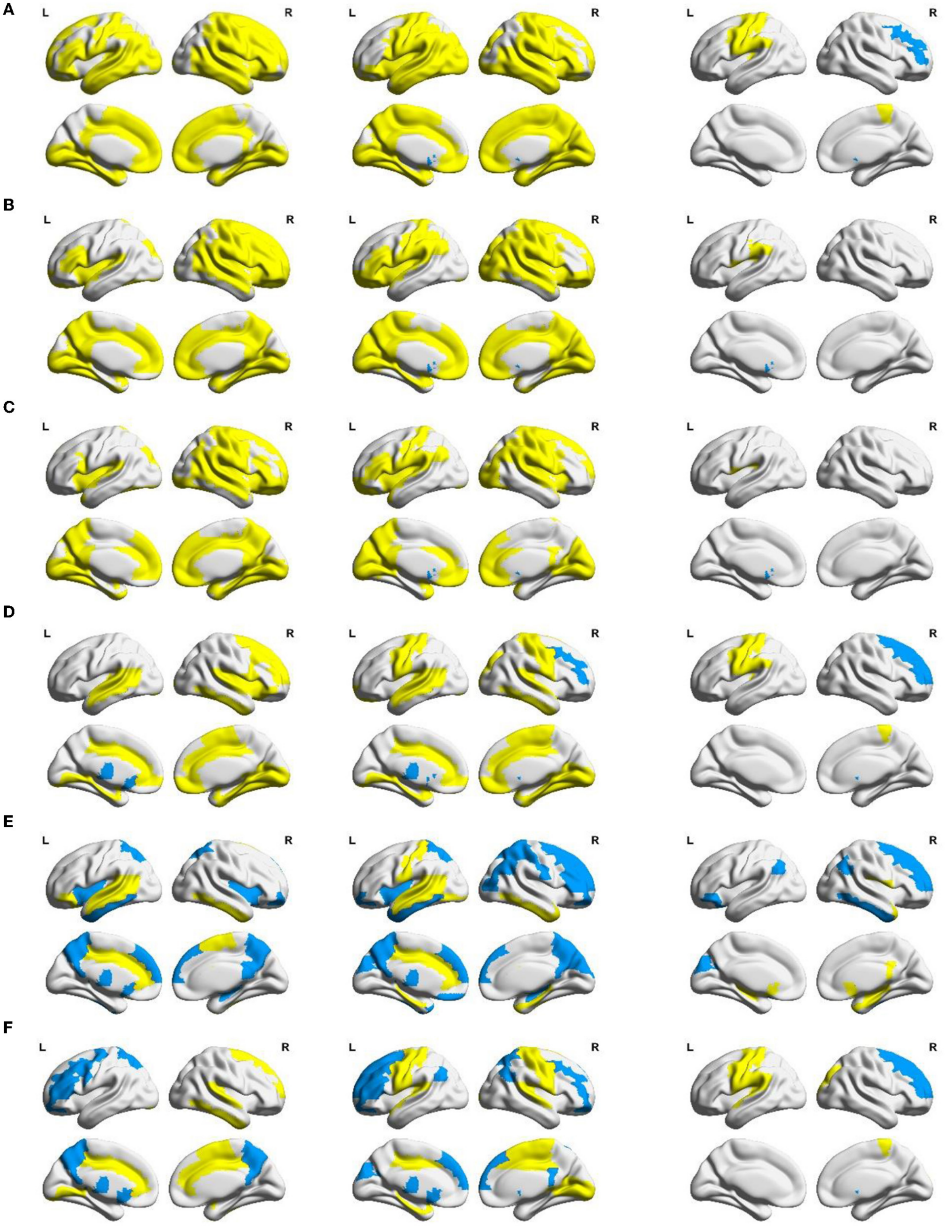
Between-group differences in AUC of local network measures: **(A)** nodal efficiency, **(B)** nodal local efficiency, **(C)** nodal clustering coefficient, **(D)** degree centrality, **(E)** betweenness centrality, and **(F)** eigenvector centrality. Yellow areas (MCS group < NC group, VS group < NC group, and VS group < MCS group) and blue areas (MCS group > NC group, VS group > NC group, and VS group > MCS group) represent local areas with significant differences.

Equally, between-group differences in each local network measure were examined using non-parametric permutation tests, which were mapped to the brain surface using BrainNet Viewer software (Xia et al., 2013) for visualization, as shown in Figure 5. In Figure 5, from left to right, the between-group differences of local network measures between the NC group and the MCS group, the NC group and the VS group, and the MCS group and the VS group are shown successively. Brain areas with significantly reduced local network measures (MCS group < NC group, VS group < NC group, and VS group < MCS group) are highlighted in yellow, and brain areas with significantly increased local network measures (MCS group > NC group, VS group > NC group, and VS group > MCS group) are marked in blue. More details on the between-group comparisons for each local network measure are provided in the Supplementary material.

Nodal efficiency, nodal local efficiency, and nodal clustering coefficient quantify the ability of neural information transmission in the node neighborhood. Compared with the NC group, nodal efficiency, nodal local efficiency, and nodal clustering coefficient were significantly reduced (*p* < 0.05) in multiple frontoparietal brain areas of MCS and VS patients. These areas mainly include the dorsolateral and medial prefrontal cortex, orbitofrontal cortex, precentral gyrus, postcentral gyrus, paracentral lobule, supplementary motor area, precuneus, inferior parietal lobule, Rolandic operculum, supramarginal gyrus, and angular gyrus. Analogous changes in nodal measures were observed in the insula, amygdala, parahippocampal gyrus, and cingulate gyrus (*p* < 0.05). Furthermore, nodal efficiency, nodal local efficiency, and nodal clustering coefficient were significantly reduced in some occipital and temporal regions (*p* < 0.05), such as the superior occipital gyrus, middle occipital gyrus, inferior occipital gyrus, cuneus, lingual gyrus, calcarine fissure and surrounding cortex, fusiform gyrus, superior temporal gyrus, middle temporal gyrus, inferior temporal gyrus, and Heschl's gyrus. Compared with the NC group, the nodal efficiency, nodal local efficiency, and nodal clustering coefficient of the bilateral caudate nucleus were significantly increased in the VS group (*p* < 0.05). Differently, there were no such changes in the MCS group yet (*p* > 0.05).

Compared with the VS group, the nodal efficiency of the right middle frontal gyrus and the right caudate nucleus decreased, and the nodal efficiency of the left precentral gyrus, left postcentral gyrus, left inferior parietal lobule, right paracentral lobule, and left Heschl's gyrus increased in the MCS group (*p* < 0.05). The nodal local efficiency and nodal clustering coefficient of the left caudate nucleus were smaller in the MCS group than those of the VS group (*p* < 0.05). The nodal local efficiency of the left Rolandic operculum and the left supramarginal gyrus was larger in the MCS group than that of the VS group (*p* < 0.05). The nodal clustering coefficient of the left Rolandic operculum was larger in the MCS group than that of the VS group (*p* < 0.05).

Nodal centrality is a key indicator to measure the influence of a node in the network. The NC group, MCS group, and VS group were compared using three common centrality measures, namely degree centrality, betweenness centrality, and eigenvector centrality. Despite the perspectives of these centrality metrics to quantify the importance of nodes being different, comparative analyses based on nodal centrality consistently showed a general readjustment of brain functional network structure in MCS and VS patients compared with the NC group. In particular, the degree centrality, betweenness centrality, and eigenvector centrality of the thalamus were significantly increased (*p* < 0.05) in MCS and VS patients, indicating an elevated status of the thalamus in the network.

As for the MCS group and VS group, the degree centrality of the right dorsolateral superior frontal gyrus, right middle frontal gyrus, and right caudate nucleus in the MCS group was lower than that in the VS group, and the degree centrality of the left precentral gyrus, left postcentral gyrus, left inferior parietal lobule, right paracentral lobule, and left Heschl's gyrus were higher than that in the VS group (*p* < 0.05). The betweenness centrality of the right dorsolateral superior frontal gyrus, right middle frontal gyrus, left orbital inferior frontal gyrus, left cuneus, bilateral angular gyrus, and right inferior temporal gyrus was lower in the MCS group than that in the VS group, and the betweenness centrality of the right Rolandic operculum, bilateral olfactory cortex, right posterior cingulate gyrus, bilateral hippocampus, right parahippocampal gyrus, right putamen, and right middle temporal gyrus was higher than that in the VS group (*p* < 0.05). The eigenvector centrality of the right dorsolateral superior frontal gyrus, right middle frontal gyrus, and right caudate nucleus was lower in the MCS group than that in the VS group, and the eigenvector centrality of the left precentral gyrus, left Rolandic operculum, right superior occipital gyrus, left postcentral gyrus, left supramarginal gyrus, right paracentral lobule, left Heschl's gyrus, and left superior temporal gyrus were higher than that in the VS group (*p* < 0.05).

**Figure 6:**
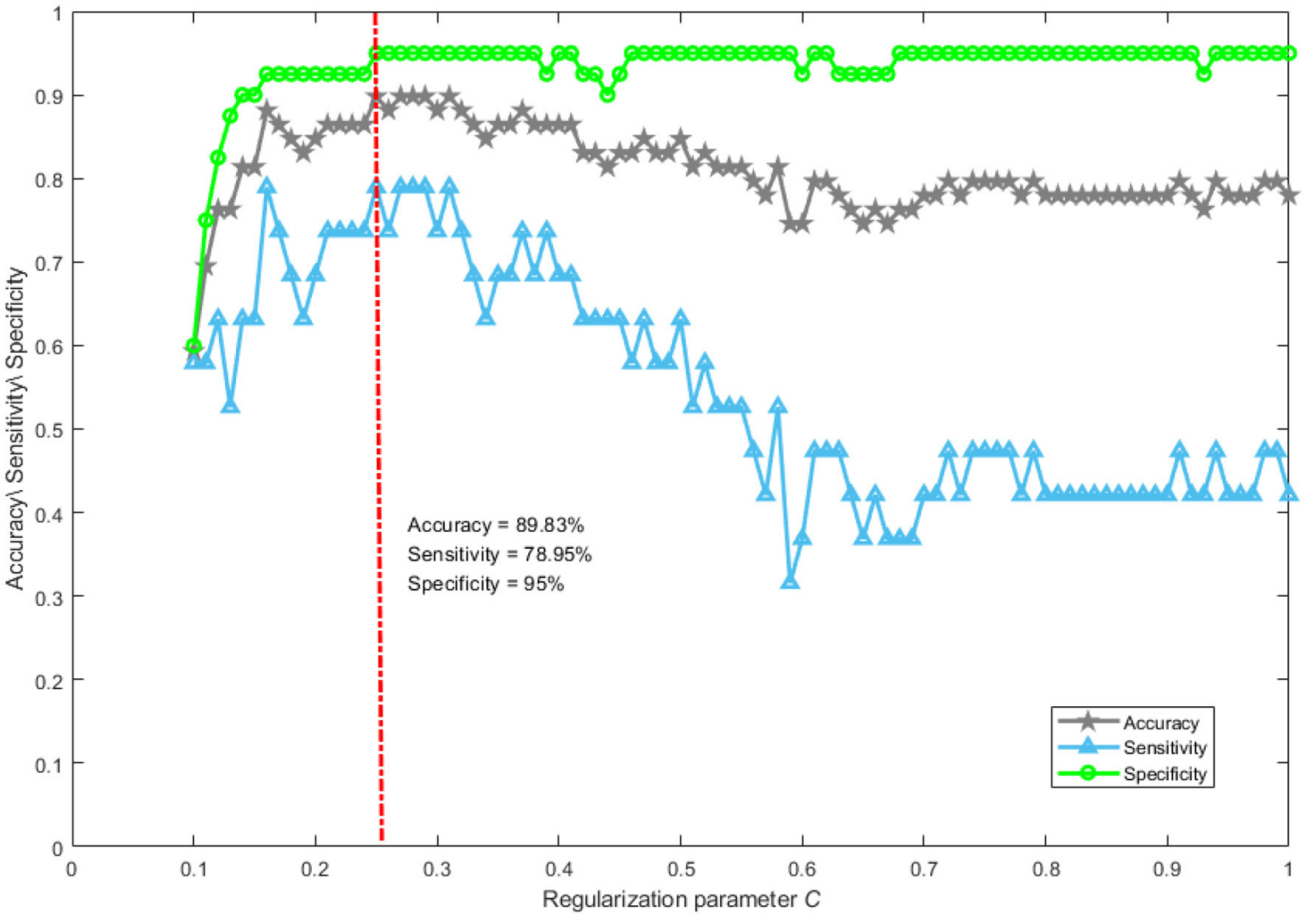
The performance of classification. Accuracy reflects the proportion of patients correctly classified into the MCS and VS groups. Sensitivity describes the proportion of patients correctly identified in the MCS group. Specificity portrays the proportion of patients correctly recognized in the VS group.

Abnormal brain activities induced by DoC can be effectively analyzed using the network mapping approach. Whether brain topological properties hold promise as a neurological marker to accurately separate MCS from VS patients has not been clearly explored. Here, a support vector machine was used to verify the distinguishing ability of brain topological characteristics. The magnification and shrinkage of the regularization coefficient λ affect the number of retained features. Given the actual mathematical expression of λ, it is more convenient to use its reciprocal *C* to control the number of retained features, which is a common practice in the field of machine learning. The smaller the parameter *C*, the larger the penalty. The performance of the classifier with the change of *C* is shown in Figure 6. We only plotted the part of *C* taking from 0.1 to 1 in steps of 0.01. From Figure 6, it can be seen that the best performance of the classifier was achieved when parameter *C* was set to 0.25. The accuracy, sensitivity, and specificity were 89.83%, 78.95%, and 95%, respectively. Notably, with the value of parameter *C* greater than 0.5, the accuracy and specificity remain approximately 70% and 95%. The sensitivity was lower than 50%. This suggests that patients who were misclassified essentially belong to the MCS group. In general, it is feasible to classify MCS patients and VS patients using network representations.

## 4. Discussion

By combining Figure 2 with Figure 3, we can see that the DoC caused a diminished dependence of most brain areas on each other. In addition, there was a similar spatial pattern of functional connectivity between the MCS group and the VS group.

Global efficiency and characteristic path length are interdependent but opposite, revealing functional integration to some extent. The increased characteristic path length and the reduced global efficiency imply that DoC occurs with a slow interaction activity of neural information and an increase in the consumption of information transfer between brain areas. Local efficiency and clustering coefficient are two metrics that describe functional separation. The reduced local efficiency and reduced clustering coefficient reflect a lower degree of network aggregation, blocking the communication between the neighbors of nodes. In other words, the brain networks of patients with impaired consciousness are loosely structured compared with normal individuals. Functional integration and separation are the basis for the emergence of cognitive behavior (Wang et al., 2021). Generally, functional integration has a positive correlation with the complexity of cognitive tasks. Functional separation determines the speed of cognitive task processing. In Figure 4, altered global network measures in DoC patients revealed weakened functional integration and weakened functional separation, which may proclaim underlying reasons for the patients failing to complete complex neuromodulation.

These reasons may be refined using the analysis of local network measures as shown in Figure 5. Frontoparietal brain areas are closely associated with higher cognitive functions (e.g., memory, emotion, movement, and language expression) (Nee, 2021). Previous studies have suggested that the frontoparietal connectivity pattern is a neural marker of behavioral awareness (Chennu et al., 2014, 2016, 2017). Insula, amygdala, parahippocampal gyrus, and cingulate gyrus are important components of the limbic system. The anterior insula and the claustrum participate in the process of consciousness through their interaction with the cortex (Critchley et al., 2004; Crick and Koch, 2005; Taylor et al., 2009; Koubeissi et al., 2014). The amygdala, parahippocampal gyrus, and cingulate gyrus play an important role in the production and expression of emotions and the storage and processing of memories. The occipital and temporal lobes are responsible for visual and auditory information processing. Abnormal nodal efficiency, nodal local efficiency, and nodal clustering coefficient of the frontoparietal brain areas, limbic system structures, occipital and temporal brain areas may account for higher cognitive dysfunctions and impaired visual and auditory activities in DoC patients.

The caudate nucleus is a part of the striatum, which is a brain region that may be involved in consciousness (Llinas et al., 2002). Some researchers have explored the possibility of using deep brain stimulation (DBS), a technique that delivers electrical impulses to specific brain areas, to treat disorders of consciousness (DoC), which are conditions that affect the level or content of consciousness in patients who have suffered a severe brain injury. One study reported that DBS increased the volume of the caudate nucleus in DoC patients (Raguz et al., 2021). However, it is controversial whether such structural changes are the result of deep brain stimulation effects or caused by recovery of consciousness. A bilateral subcortical (thalamus and caudate nucleus) and cortical (frontal–temporal–parietal) network exhibits metabolic damage in non-traumatic and traumatic MCS patients (Bruno et al., 2012). According to the presence or absence of language-related consciousness signs, MCS can be divided into MCS- and MCS+. Compared with MCS- patients, brain areas such as the precuneus, thalamus, left caudate nucleus, and temporal/angular cortices associated with semantics showed less hypometabolism and/or larger gray matter volume in MCS+ patients (Aubinet et al., 2019). Therefore, it may be reasonable to speculate that compared with the NC group the increased nodal efficiency, nodal local efficiency, and nodal clustering coefficient in the caudate nucleus of the VS group as well as the non-significant changes in these indicators of the MCS group may be caused by activated brain compensatory functions.

In terms of nodal efficiency, the topological differences between the MCS and VS groups were mainly in the frontoparietal lobe. This was consistent with comparisons of synaptic activity measured indirectly in the resting state based on changes in glucose metabolic rate or cerebral blood flow in various brain regions of MCS and VS patients (Stender et al., 2015). The Heschl's gyrus is located in the primary auditory cortex. Compared with the VS group, the increased nodal efficiency of Heschl's gyrus in MCS patients may be related to their residual external consciousness capacity (auditory localization). Although there is some ability in the functional connectivity of the visual and auditory cortex to differentiate MCS and VS patients, the activation of the visual and auditory cortex does not play a decisive role in the emergence of consciousness. Notably, as the level of consciousness increases, neural information interactions between the caudate nucleus and other brain areas were inhibited in a local scope, as opposed to Rolandic operculum and supramarginal gyrus, as derived from the corresponding changes in nodal efficiency, nodal local efficiency, and nodal clustering coefficient. Rolandic operculum and supramarginal gyrus are situated in Wernicke's area, which controls language understanding. Clinical evidence shows that many patients with MCS can respond to speech or follow verbal commands (Aubinet et al., 2019). This may garner benefit from the preservation of the neural information communication capacity in speech-related cortices.

The altered node centrality captured the brain network structure of DoC patients reorganized on a large scale. Particularly, the importance of the thalamus in the network was improved. The increased degree of centrality illustrates that the thalamus has a stronger coupling relationship with its neighbors. The increased betweenness centrality suggests that the thalamus has enhanced communication control over other brain areas. The increased eigenvector centrality implies that the neighbors of the thalamus also have a great influence on the whole network. The thalamus is considered to be the switch of consciousness (Lemaire et al., 2022). A large number of studies suspect that reduced cortical–thalamocortical functional connectivity is key to the loss of consciousness (Malekmohammadi et al., 2019). Our results may also need to be explained by the compensatory mechanism of the brain functional network. Furthermore, similar to the comparison based on nodal efficiency, the centrality differences between MCS and VS patients were mainly in frontoparietal brain areas.

When the value of parameter C is greater than 0.5, the sensitivity was lower than 50%. Such a phenomenon is not surprising, mainly for the following reasons. Our classification task suffers from a severe class imbalance, which causes the classifier to learn a priori information of sample proportion. Thus, the actual classification is focused on the majority class. In addition, many redundant features are retained when the value of parameter C is controlled above 0.5. This is a disaster for support vector machines if the features are of high dimensionality and contain a lot of redundancy. At this point, even if the class imbalance problem is improved by an oversampling technique, the classification performance is still poor.

## 5. Conclusion

hThe network mapping method can effectively recognize the specific brain areas related to DoC, providing a new window to explain the pathological mechanism of DoC. Network characterization is effective in distinguishing MCS from VS patients. Nevertheless, there were some limitations in this study. First, our study was based on a small size of samples with unbalanced classes. More reliable and convincing results need to be supplemented by collecting sufficient samples with unbalanced classes in future. Second, only the AAL90 brain template was utilized to segment the brain to define the network nodes. Some new findings may be obtained using a more refined brain segmentation scheme. Next, the performance of other classifiers, especially the emerging deep learning algorithms, has not yet been discussed. Nevertheless, these algorithms require a large amount of data for training. Finally, the human brain achieves goal-directed behavior by dynamically regulating neuronal activities. In the future, it is essential to construct dynamic brain functional networks in patients with DoC.

## Data availability statement

The data analyzed in this study is subject to the following licenses/restrictions: In this study, data are not publicly available but are available from the corresponding author on reasonable request. Requests to access these datasets should be directed to he_jianghong@sina.cn.

## Ethics statement

The studies involving human participants were reviewed and approved by the Beijing Tiantan Hospital, Capital Medical University. The patients/participants provided their written informed consent to participate in this study. Written informed consent was obtained from the individual(s) for the publication of any potentially identifiable images or data included in this article.

## Author contributions

YD proposed conceptualization, methodology, completed a formal analysis, and wrote the original draft of the manuscript. YY, XC, XG, and JH designed the experiment and collected data. YY, YD, QH, SW, and FD reviewed and edited the manuscript. All authors contributed to the article and approved the submitted version.
