# Supplementary material for: Altered brain functional connectivity in vegetative state and minimally conscious state
Source: Front Aging Neurosci. 2023 Jun 29;15:1213904. doi: 10.3389/fnagi.2023.1213904 (PMC10352323; doi:10.3389/fnagi.2023.1213904)
Supplement: Supplementary file 1 [file Table_1.DOCX]

Supplementary Material

Altered Brain Functional Connectivity in Vegetative State and Minimally Conscious State

Yi Yang1,4,5,6†, Yangyang Dai2†, Qiheng He1, Shan Wang3, Xueling Chen1, Xiaoli Geng1, Jianghong He1*, Feng Duan2*

*** Correspondence:**

Duan Feng, Email: [duanf@nankai.edu.cn](mailto:duanf@nankai.edu.cn)

He Jianghong, Email: [he_jianghong@sina.cn](mailto:he_jianghong@sina.cn)

In this study, patients were evaluated with CRS-R twice weekly (or more) within two weeks prior to MRI scanning. The highest CRS-R scores were regarded as diagnostic criteria and presented in the Table S1 below.

Table S1. The specific demographic and clinical information for DoC patients

| Patient alias | Gender | Age(years) | Diagnose | Etiology | Time to MRI(Months) | CRS-R score |
| --- | --- | --- | --- | --- | --- | --- |
| Sub001 | M | 48 | MCS | Intracranial Hemorrhage | 11 | 12(315102) |
| Sub002 | F | 55 | MCS | stroke | 3 | 9(312102) |
| Sub003 | M | 39 | MCS | Intracranial Hemorrhage | 9 | 9(222102) |
| Sub004 | M | 23 | MCS | Traumatic Brain Injury Caused by a fall | 5 | 7(103002) |
| Sub005 | M | 54 | MCS | stroke | 3 | 8(113102) |
| Sub006 | M | 23 | MCS | Cardiopulmonary Arrest | 3 | 10(232102) |
| Sub007 | F | 27 | MCS | Traumatic Brain Injury | 10 | 12(332103) |
| Sub008 | F | 64 | MCS | Traumatic Brain Injury | 2 | 6(103101) |
| Sub009 | M | 22 | MCS | Traumatic Brain Injury Caused by a Traffic Accident | 6 | 9(132102) |
| Sub010 | M | 28 | MCS | Traumatic Brain Injury | 9 | 18(355113) |
| Sub011 | M | 53 | MCS | Hemorrhage of Brain Stem | 7 | 11(332102) |
| Sub012 | M | 44 | MCS | Intracranial Hemorrhage | 2 | 9(132102) |
| Sub013 | M | 53 | MCS | stroke | 4 | 7(102202) |
| Sub014 | F | 70 | MCS | stroke | 3 | 8(113102) |
| Sub015 | F | 28 | MCS | Traumatic Brain Injury | 1 | 15(335103) |
| Sub016 | F | 30 | MCS | Traumatic Brain Injury Caused by a Traffic Accident | 3 | 11(133202) |
| Sub017 | M | 30 | MCS | Traumatic Brain Injury Caused by a Traffic Accident | 2 | 11(134102) |
| Sub018 | F | 38 | MCS | Amniotic Fluid Embolism,  Cardiopulmonary Arrest | 2 | 6(111102) |
| Sub019 | F | 32 | MCS | Traumatic Brain Injury | 12 | 7(102202) |
| Sub020 | F | 63 | VS | Intracranial Hemorrhage | 1 | 7(112102) |
| Sub021 | F | 30 | VS | Amniotic Fluid Embolism,  Cardiopulmonary Arrest | 1 | 7(112102) |
| Sub022 | M | 43 | VS | stroke | 7 | 6(111102) |
| Sub023 | M | 47 | VS | Amniotic Fluid Embolism,  Cardiopulmonary Arrest | 1.5 | 6(111102) |
| Sub024 | M | 31 | VS | stroke | 3 | 6(102102) |
| Sub025 | M | 53 | VS | stroke | 6 | 7(112102) |
| Sub026 | F | 29 | VS | Septic Shock | 2 | 4(002002) |
| Sub027 | M | 61 | VS | Traumatic Brain Injury Caused by a fall | 3 | 5(002102) |
| Sub028 | F | 24 | VS | Anesthetic Accident,  Hypoxic-ischemic Encephalopathy | 3 | 5(102002) |
| Sub029 | F | 50 | VS | Traumatic Brain Injury | 3 | 7(112102) |
| Sub030 | M | 66 | VS | Intracranial Hemorrhage | 1 | 5(112100) |
| Sub031 | M | 48 | VS | Electrical Injury,  Hypoxic-ischemic Encephalopathy | 4 | 7(112102) |
| Sub032 | M | 53 | VS | Hemorrhage of Brain Stem | 4 | 5(112100) |
| Sub033 | M | 31 | VS | Cardiopulmonary Arrest | 2 | 5(002102) |
| Sub034 | F | 53 | VS | Hemorrhage of Brain Stem | 3 | 5(112100) |
| Sub035 | M | 15 | VS | Traumatic Brain Injury | 3 | 5(112100) |
| Sub036 | F | 39 | VS | stroke | 11 | 6(102102) |
| Sub037 | M | 51 | VS | Amniotic Fluid Embolism,  Cardiopulmonary Arrest | 2 | 7(103102) |
| Sub038 | F | 35 | VS | Hypoxic-ischemic Encephalopathy | 3 | 6(102102) |
| Sub039 | M | 23 | VS | Traumatic Brain Injury | 10 | 5(101102) |
| Sub040 | M | 53 | VS | Traumatic Brain Injury | 15 | 7(112102) |
| Sub041 | M | 45 | VS | Cerebral Infarction | 3 | 7(112102) |
| Sub042 | F | 32 | VS | stroke | 3 | 7(112102) |
| Sub043 | F | 71 | VS | Traumatic Brain Injury | 2 | 7(112102) |
| Sub044 | M | 53 | VS | Hypoxic-ischemic Encephalopathy | 2 | 3(002001) |
| Sub045 | M | 27 | VS | Traumatic Brain Injury | 4 | 6(101202) |
| Sub046 | M | 39 | VS | stroke | 4 | 2(002000) |
| Sub047 | F | 56 | VS | Cerebral Infarction | 5 | 7(112102) |
| Sub048 | M | 54 | VS | stroke | 4 | 7(112102) |
| Sub049 | M | 53 | VS | stroke | 2 | 4(011002) |
| Sub050 | F | 31 | VS | Hypoxic-ischemic Encephalopathy | 1 | 6(102102) |
| Sub051 | M | 28 | VS | Amniotic Fluid Embolism,  Cardiopulmonary Arrest | 4.5 | 3(001002) |
| Sub052 | F | 69 | VS | Intracranial Hemorrhage | 4 | 6(102102) |
| Sub053 | F | 41 | VS | stroke | 2 | 6(112002) |
| Sub054 | F | 41 | VS | Anoxia | 2 | 5(002102) |
| Sub055 | M | 43 | VS | Epileptic Seizure | 2 | 6(102102) |
| Sub056 | M | 43 | VS | Traumatic Brain Injury | 4 | 5(112100) |
| Sub057 | F | 43 | VS | Hypoxic-ischemic Encephalopathy | 2 | 7(112102) |
| Sub058 | M | 33 | VS | stroke | 1 | 5(002102) |
| Sub059 | F | 18 | VS | Traumatic Brain Injury | 1 | 7(112102) |
